# TAK-653 Reverses Core Depressive Symptoms in Chronic Stress-Induced Monkey Model

**DOI:** 10.3390/biomedicines13061389

**Published:** 2025-06-05

**Authors:** Ling Li, Zhiting Zhang, Xinhe Liu, Mengni Zhou, Shenglin Wen, Ji Dai

**Affiliations:** 1 Department of Psychology, The Fifth Affiliated Hospital, Sun Yat-Sen University, Zhuhai 519000, China; liling83@mail.sysu.edu.cn; 2 Shenzhen Technological Research Center for Primate Translational Medicine, Shenzhen-Hong Kong Institute of Brain Science, Shenzhen Institutes of Advanced Technology, Chinese Academy of Sciences, Shenzhen 518055, China; 3 CAS Key Laboratory of Brain Connectome and Manipulation, The Brain Cognition and Brain Disease Institute, Shenzhen Institutes of Advanced Technology, Chinese Academy of Sciences, Shenzhen 518055, China; 4 Guangdong Provincial Key Laboratory of Brain Connectome and Behavior, Shenzhen Institutes of Advanced Technology, Chinese Academy of Sciences, Shenzhen 518055, China; 5 School of Software, Taiyuan University of Technology, Taiyuan 030024, China; 6 University of Chinese Academy of Sciences, Beijing 100049, China

**Keywords:** major depressive disorder, TAK-653, chronic unpredictable mild stress, cytokines, monkey

## Abstract

**Background:** Major Depressive Disorder represents a prevalent and critical mental health issue that highlights the pressing need for innovative therapeutic solutions. Recent research has identified dysfunction within the glutamate system as a crucial element influencing both the onset and management of depressive symptoms. Although TAK-653 is a new positive allosteric modulator of AMPA receptors, its effects have not been rigorously examined in models of depression in primates. **Methods:** To assess its potential antidepressant properties, a chronic unpredictable mild stress protocol was implemented over 12 weeks to create a monkey model of depression, followed by a two-week treatment period with TAK-653. **Results:** Behavioral evaluations showed that following stress exposure, the monkeys exhibited reduced motivation for food, increased huddling, diminished movement, and a tendency to remain at the lower levels of their enclosure. They also displayed heightened anxiety in response to external stimuli. Plasma analyses indicated higher levels of cortisol, IL-6, and IL-8 in the stressed monkeys compared to baseline readings, confirming the efficacy of the stress-inducing protocol. Post-treatment with TAK-653 resulted in significant improvements, such as enhanced motivation for food, less huddling behavior, greater activity, and a move towards the upper areas of the enclosure. Additionally, the plasma analysis revealed a marked decrease in cortisol and IL-6 levels, along with an increased expression of BDNF. **Conclusions:** These findings indicate that TAK-653 effectively alleviates depression-like behaviors in nonhuman primate models, thereby paving the way for a promising new strategy in the treatment of depression.

## 1. Introduction

Depression stands as a prevalent mental health disorder, characterized by key symptoms such as a persistently low mood, reduced motivation, and diminished interest or pleasure in activities [1,2]. The World Health Organization has reported that around 5% of adults around the globe are affected by depression, with projections indicating that, by 2030, it will emerge as the most widespread health condition worldwide. The exact causes of Major Depressive Disorder (MDD) remain uncertain, and current treatment approaches largely hinge on the monoamine theory. However, traditional monoaminergic antidepressants often exhibit delayed effects, high rates of recurrence, numerous side effects, and prove ineffective for over one-third of patients, who are categorized as having treatment-resistant depression (TRD) [3]. Given the limited effectiveness of existing medications, there is a pressing need for innovative antidepressants that deliver quick, strong, and lasting therapeutic benefits for individuals suffering from depression, including those with TRD.

Ketamine, an N-methyl-D-aspartate receptor (NMDAR) antagonist, has ushered in significant advancements in mood disorder research, particularly due to its rapid and potent antidepressant properties seen in patients [4]. Extensive investigation into ketamine’s mechanism of action has led to the widely accepted “disinhibition hypothesis” [5]. This hypothesis postulates that ketamine selectively inhibits NMDARs on GABAergic interneurons, resulting in an unexpected surge of glutamate release that activates α-amino-3-hydroxy-5-methyl-4-isoxazolepropionic acid receptors (AMPAR) [6]. The subsequent activation of AMPAR fosters the release of brain-derived neurotrophic factor (BDNF) and initiates the mTOR signaling pathway. In rodent studies, it has been observed that pre-treatment with the AMPAR inhibitor NBQX significantly lowered BDNF and mTOR levels in both the prefrontal cortex and hippocampus, negating the antidepressant effects of ketamine. Conversely, administering an AMPAR agonist prior to ketamine treatment raised BDNF and mTOR levels, thereby amplifying ketamine’s antidepressant effects [7]. Collectively, these findings underscore the pivotal role that AMPA receptor-mediated BDNF release and mTOR-related cellular signaling play in the swift antidepressant action of ketamine. Nonetheless, the potential for adverse effects, including psychotomimetic experiences, dissociation, and the risk of misuse associated with ketamine, could limit its broader application [8]. Consequently, targeting the AMPA receptor presents a promising avenue for achieving enhanced and safer therapeutic outcomes for those suffering from MDD.

AMPAR activators comprise a category of substances known as AMPAR agonists and potentiators. A notable concern arises with the systemic administration of AMPAR agonists, which inadvertently activates AMPARs in a non-selective manner, significantly increasing the risk of seizures while demonstrating a bell-shaped dose–response relationship [9,10]. Various AMPAR potentiators, including CX516, CX691, CX717, Org26576, LY451395, S18986, and S47445, have been subjected to clinical assessments aimed at addressing neuropsychiatric conditions, such as cognitive deficits related to Alzheimer’s disease or schizophrenia, depressive symptoms, and attention deficit/hyperactivity disorder [11]. Thus, AMPAR potentiators that exhibit minimal agonistic properties have the potential to introduce a new class of fast-acting antidepressants. TAK-653, a novel AMPAR potentiator characterized by its low agonistic activity, has shown promise in preclinical studies [12]. In primary neuronal cultures derived from rats, TAK-653 was observed to activate downstream signaling pathways aligned with those activated by ketamine. Furthermore, robust antidepressant-like effects were documented in the rat Resistant-to-Submissive Behavior Model [12]. Investigations in healthy human subjects confirm the safety of TAK-653 [13,14], and a Phase II clinical trial has reported its efficacy in improving depression scores at select doses (ClinicalTrials.gov identifier: NCT05203341). To deepen our understanding of the drug’s mechanism and to inform the design of future clinical trials, it is essential to study TAK-653 in an animal model that closely mirrors human physiology.

Stress is recognized as a significant contributor to the development of depression [15]. Chronic exposure to prolonged stress can impair the normal functioning of the hypothalamic–pituitary–adrenal (HPA) axis, resulting in elevated cortisol levels. Additionally, stress triggers the activation of microglia within the brain, leading to an increase in the release of pro-inflammatory cytokines such as IL-6, IL-8, and TNF-α; these cytokines subsequently influence neurogenesis and synaptic plasticity [16]. For an extended period, depression has been viewed as a condition associated with immune hyperactivation, where elevated levels of pro-inflammatory cytokines are critical in the context of neuroinflammation, significantly impacting the manifestation and severity of depressive symptoms [17,18]. The chronic unpredictable mild stress (CUMS) model is particularly effective in mimicking the psychosocial stress factors that contribute to human depression [19]. Nonhuman primates (NHPs) share numerous similarities with humans, including the structural and functional characteristics of the central nervous system, aspects of neurodevelopment, social behaviors, emotional regulation, and stress responses, which provide them with unique advantages in modeling MDD [20,21]. NHPs possess a genome that is 93% identical to that of humans. This substantial evolutionary similarity renders NHP models exceptionally valuable for translational research. Empirical evidence demonstrates that NHP models exhibit markedly greater predictive validity for clinical outcomes in humans when compared to rodent models [22]. Consequently, this study adopts the CUMS model typically employed in rodent MDD research to establish a primate MDD model [23]. This approach aims to investigate the antidepressant effects of TAK-653 and explore its underlying mechanisms. The findings from this research are expected to advance the understanding of TAK-653 as an innovative antidepressant while further illuminating the role of AMPA receptors in MDD.

## 2. Materials and Methods

### 2.1. Animals

The experiment was conducted using three male cynomolgus monkeys (*Macaca fascicularis*), weighing 7.4 kg, 8 kg, and 9 kg, respectively, all aged 7 years at the beginning of the experiment. The decision to limit the study to only three subjects was guided by strict adherence to the 3R ethical framework of animal research—Replacement, Reduction, and Refinement. This sample size represents a careful balance between ensuring sufficient statistical validity to achieve the study’s objectives and upholding the ethical responsibility to minimize the use of animals. These monkeys were obtained from Jingang Biotech (Hainan, China) and individually housed in a facility accredited by the Association for Assessment and Accreditation of Laboratory Animal Care (AAALAC) [24,25]. The environment was carefully controlled, maintaining humidity levels between 40 and 70% and temperatures from 20 to 26 °C, accompanied by a 12 h light–dark cycle (lights on from 7:00 AM to 7:00 PM) [26]. Each monkey received routine care from a veterinarian, was provided with fresh fruits, vegetables, and a sufficient number of nutrient-rich monkey biscuits, along with unrestricted access to clean water. The monkeys were accommodated in individual cages (110 × 110 × 90 cm), with the flexibility to adjust their activity space via an external lever mechanism. The cages were designed to be open only at the front, while the remaining sides were secured with metal panels. All experimental procedures conformed rigorously to the “Guide for the Care and Use of Laboratory Animals” established by the National Institutes of Health (NIH) and received approval from the Institutional Animal Care and Use Committee (IACUC) at the Shenzhen Institutes of Advanced Technology, Chinese Academy of Sciences (Approval Number: SIAT-IACUC-230904-NS-DJ-A2316). Upon completion of the experiment, the monkeys were relocated to group-housing rooms. Throughout the entire study duration, all animals maintained good health and survived without incident.

### 2.2. Experimental Procedures

**Figure 1:**
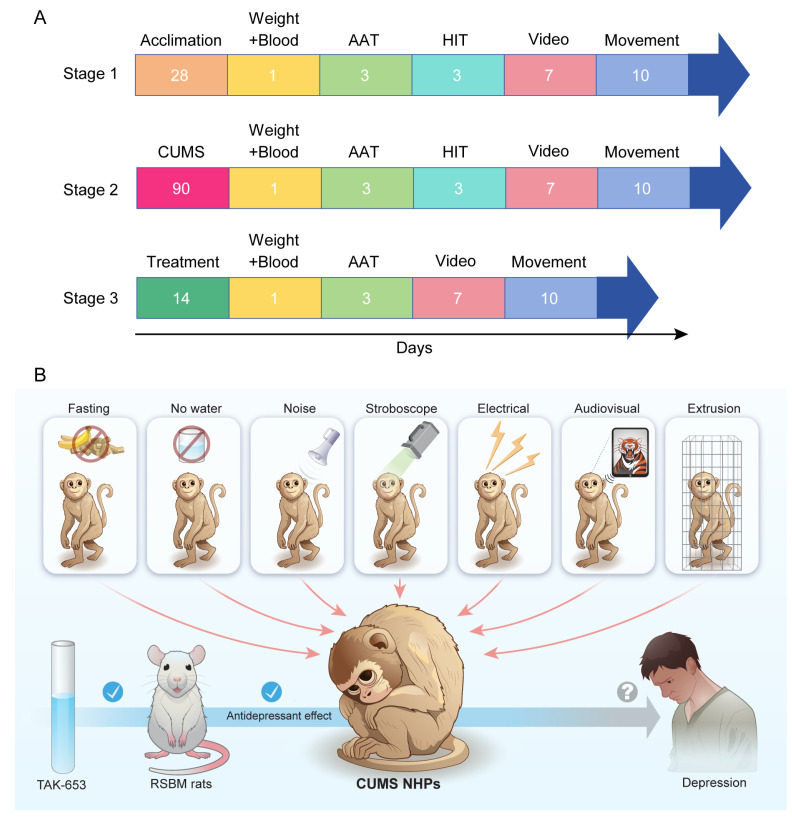
Schematic representation of the experimental design. (**A**) The general timeline of the experiment. (**B**) The Chronic Unpredictable Mild Stress (CUMS) protocol was utilized on cynomolgus monkeys to create a model of Major Depressive Disorder (MDD). Subsequently, the antidepressant efficacy of TAK-653 was assessed in these depressed subjects, laying the groundwork for potential future clinical applications.

The experiment included the acclimation, CUMS, and treatment stages (Figure 1A). Before commencing the CUMS experiment, the three monkeys underwent a 4-week acclimation period in the designated monkey room to adapt to their housing conditions. Throughout this acclimation phase, the experimental operator provided the monkeys with food and water, as well as fruits and vegetables. Additionally, the monkeys were trained to sit in a special monkey chair and to familiarize themselves with various procedures within the room, including the presence of cameras and other apparatuses. This preparatory process aimed to alleviate potential stress responses that could interfere with the experiments once they began.

Following this acclimatization, the CUMS experimental phase was conducted over a span of three months. The stressors utilized in this study closely mirrored those used in a prior monkey experiment [27], themselves adapted from earlier rodent research [28,29], and included the following elements: fasting for 24 h, water deprivation for 12 h, exposure to noise (80 dB via a buzzer for 12 h), strobe light exposure for 12 h, inescapable foot shocks (1.0–1.25 mA for durations of 3–5 s, with 10 s intervals repeated 3–4 times), audiovisual stimulation, and spatial restriction lasting 4 h (Figure 1B, Table 1). All stress conditions were meticulously calibrated and strictly implemented to prevent any undue pain or distress. Each day, two different stressors were applied, with careful measures taken to avoid repeating the same stressor on consecutive days. Neither food deprivation nor water restriction was ever imposed on the same day, nor were these conditions applied on back-to-back days. Additionally, the electrical stimuli were self-tested to guarantee the administration of the mildest effective shocks. The audiovisual stimulation primarily featured footage of large predators and birds of prey, such as tigers, lions, eagles, and hawks, performing natural behaviors like roaring, running, chasing, and attacking their prey. Accompanying sound effects included the various roars and calls of these predators, played at a volume of approximately 80 dB. The video content was updated monthly. During the initial two months, the duration of the video stimulation was set at 2.5 h, which increased to 4 h in the third month.

Throughout the stressor interval, it is imperative to rigorously assess the sufficiency of blood circulation to the limbs and to meticulously detect any possible injuries to the skin and mucous membranes. Each monkey underwent a thorough physical examination following every stimulation session. Upon completion of the CUMS period, the recorded body weights of the three monkeys were 7.6 kg, 8.75 kg, and 9.3 kg, respectively, each exceeding their initial baseline measurements.

### 2.3. Measurement of Blood Cortisol and Inflammatory Cytokines

Blood samples were collected from monkeys between 9:00 and 9:30 a.m. at baseline, biweekly throughout the stress period, at the conclusion of stress exposure, and following TAK-653 treatment. Venous blood was drawn from the cubital vein into 5 mL vacuum tubes containing lithium heparin. Subsequently, all samples were centrifuged at 3000 rpm for 15 min, after which the plasma was aliquoted into 100 µL portions and stored at −80 °C. Using a double-blind approach, the levels of cortisol in the blood were determined using various commercially available kits, including the Monkey Cortisol ELISA Kit (EMK0084, FineTest, Wuhan, China), Monkey IL-4 ELISA Kit (LEMK040-2S, LAIZEE, Shanghai, China), Monkey IL-6 ELISA Kit (LEMK060-2S, LAIZEE, Shanghai, China), Monkey IL-8 (Interleukin 8) ELISA Kit (EMK0042, FineTest, Wuhan, China), Monkey IL-10 ELISA Kit (LEMK100-2S, LAIZEE, Shanghai, China), Monkey TNF-α ELISA Kit (LEMK810-2S, LAIZEE, Shanghai, China), Monkey IL-1β ELISA Kit (LEMK012-2, LAIZEE, Shanghai, China), and Monkey BDNF ELISA Kit (EP20RB, Thermofisher, Waltham, MA, USA) via enzyme-linked immunosorbent assay. Each blood sample underwent duplicate testing, and the mean of the two measurements was employed for subsequent statistical analysis.

### 2.4. Behavioral Tests

Following the acclimation period, baseline assessments were conducted for the Attempt for Apple Test (AAT) [30], as well as the Human Intruder Test (HIT) and baseline behavior recordings within a specially designed observing cage. In addition, a motion tracker was attached to the animals’ collars. The AAT was performed at baseline, immediately after stress exposure, and post-TAK-653 administration. Initially, apple pieces were cut into strips and suspended from the cage bars. The monkeys’ interactions were observed for a duration of 15 min, tallying the number of attempts they made to reach for the apple strips. However, while the monkeys reached for the apple strips during the initial minutes, they eventually ceased their efforts despite being hungry. This prompted the experimenter to revise the AAT methodology. Apples were instead cut into pieces comparable in size to the monkey biscuits provided during regular feeding times. For each trial, 30 pieces of monkey biscuit were prepared alongside 30 apple cubes. A rectangular wooden board with two circular indentations on either end was positioned in front of the monkey enclosure to facilitate access to the food. During intervals between feedings, the apple cubes and monkey biscuits were alternated in the indentations, allowing the monkeys to make their selection. Once one item was chosen, the other would be promptly removed. The placement of the apple and biscuit was switched for subsequent trials. To ensure the validity and consistency of our results, each method was repeated across three consecutive days, conducting one trial daily. In the context of the AAT, a lower rate of selecting apple cubes in comparison to monkey biscuits was interpreted as a sign of anhedonia, a significant indicator of MDD.

HITs were conducted both at the baseline and following the stress exposure period [31]. In each HIT session, a ten-minute high-resolution video was recorded. The initial two minutes of the assessment were carried out with no one entering the housing room, to establish a baseline condition. In the subsequent two minutes, an individual who had no prior interaction with the subject before the test, the “intruder”, entered the housing room, positioning themselves approximately 100 cm in front of the monkey cage and facing the monkey from the right. During the third two-minute interval, the intruder turned 90 degrees to the right, directly confronting and staring at the monkey. In the fourth interval, the intruder rotated another 180 degrees, turning their back to the monkey. For the fifth two-minute segment, the intruder once again faced the monkey cage, maintained eye contact with the monkey, and waved both hands. Then, the intruder left the room. After the testing process, two additional experimenters, who had not interacted with the monkeys, reviewed the videos and recorded the duration of various behaviors exhibited during each two-minute segment. These behaviors included freezing, skin scratching, yawning, displaying signs of fear, retreating to the bottom of the cage, slow movements, shaking the cage, swaying, grooming, and lip smacking. Among these actions, freezing, scratching, yawning, and showing signs of fear were categorized as anxiety-related behaviors (Table 2). To improve the reliability of our findings, each cycle of the test was carried out over three consecutive days, with one trial each day. In the HIT, the total duration of the four anxiety-related behaviors observed in each two-minute segment served as a quantitative metric to evaluate anxiety-like responses and reactions to external stimuli in the monkey subjects.

A custom-made observing cage (100 × 100 × 100 cm) was placed in the designated recording room to monitor the spontaneous activity of monkeys both at baseline and following stress exposure [32]. The cage’s top and front were constructed with tempered glass to facilitate direct video recording. Two cameras were utilized, one for capturing a top-down view (covering the x and y dimensions) and another for a side view (encompassing the x and z dimensions). The commercial system (Vigie Primate, ViewPoint, Inc., Lyon, France), previously applied in other studies for analyzing locomotor behavior in NHPs [33,34], was implemented to evaluate the subjects’ activities and extract movement data from the recorded footage. This video tracking system operated at a sampling frequency of 25 Hz, with both the top and side views synchronized. Movement tracking data for all three axes (x, y, and z) were normalized on a scale from 0 to 100 cm, where the x-axis indicated lateral movement, the y-axis represented forward and backward movement, and the z-axis depicted vertical movement. The videos, which documented the monkeys’ behaviors, were recorded for a minimum of 180 min. Customized codes were employed to reconstruct and quantify the movement trajectories from the collected data. To enhance the objectivity and precision of the behavioral assessment, an automated neurobehavioral monitoring and analysis tool was also utilized (PrimateScan, CleverSys, Inc., Reston, VA, USA) to track and analyze the monkeys’ behaviors throughout the complete experimental timeline.

According to the ethogram for *Macaca fascicularis* [35], nine distinct behaviors of monkeys housed in individual cages have been identified: huddling posture, locomotion, stereotypic behaviors, environmental responses, climbing, self-grooming, ball play, observing, and sitting (Table 3). The huddling posture, characterized by self-clasping with the head positioned at or below the shoulders while the monkey is awake, is recognized as a primary indicator of depressive mood in these animals [36]. Previous research has indicated that macaques exhibiting signs of depression tend to have diminished physical activity, specifically less time spent walking within their enclosures [37,38]. Additionally, other studies have further corroborated this finding by noting a reduction in locomotion time among depressed macaques [39]. To accurately measure the monkeys’ daily movement, a motion tracking device (GT9X-BT, ActiGraph, Pensacola, FL, USA) was affixed to them in their home cages. This compact device, measuring 2 × 2 × 1 cm, was securely housed within an aluminum case and attached to the animals’ collars for a minimum duration of one week. The monitor is equipped with a magnetometer that detects movement direction by assessing the local magnetic field strength relative to the Earth’s North Pole. The collected data were managed, analyzed, and visualized using the professional software (ActiLife 7), where customized codes were employed to quantify the daily step count from the recorded movement data.

### 2.5. TAK-653 Administration

TAK-653, identified as 9-[4-(cyclohexyloxy)phenyl]-7-methyl-3,4-dihydropyrazino[2,1-c][1,2,4]thiadiazine 2,2-dioxide, was acquired from MedChemExpress (MCE) in Monmouth Junction, NJ, USA, under Catalog No. HY115864, with a purity level of 99%. Previous research has indicated that administering TAK-653 orally at doses between 0.03 and 50 mg/kg to rats yields significant antidepressant effects [40,41]. Furthermore, a dose of 0.06 mg/kg in monkeys has been associated with cognitive enhancement [41]. To establish the appropriate dosage for monkeys, the body surface area ratio between monkeys and rats was calculated using the equation: $Ratio=\frac{Km}{Kr}\times ({\frac{Wm}{Wr})}^{\frac{2}{3}}$, where *Km* and *Kr* represent the body size coefficients for monkeys and rats, respectively, and *Wm* and *Wr* denote their respective weights. This formula is a modification of the Meeh’s formula [42], with empirical values assigned as *Km* = 0.111 and *Kr* = 0.09. Assuming the weights of the monkey and rat are 9 kg and 0.2 kg, respectively, the resulting ratio is 15.99. Subsequently, the monkey dosage was determined by the following equation: $Dose_{monkey}={Dose}_{rat}\times Ratio\times \frac{\;Wr}{Wm}$. Given that an effective dose in rats is 1 mg/kg [40], the equivalent dose for a 9 kg monkey calculates to 0.346 mg/kg. In the present study, the TAK-653 reagent was precisely weighed each morning. The monkey biscuit powder was combined with warm water to create a dough consistency, which was subsequently shaped into small pieces approximately 1 cm in diameter. Each dough portion contained the TAK-653 reagent in its center, which was administered to the monkeys. Given that peak plasma concentrations of TAK-653 are reached within 1 to 2 h following an oral dose of 1 mg/kg in rats, the monkeys were then moved to observing cages for behavioral video recording 30 min post-feeding. After the treatment period, the body weights for the three monkeys were 7.65, 8.5, and 9.2 kg, respectively.

### 2.6. Statistical Analysis

Statistical analyses were conducted utilizing IBM SPSS Statistics for Windows, Version 27 (IBM Corp., Armonk, NY, USA). The analyses of AAT, cortisol, IL-1β, IL-4, IL-6, IL-8, IL-10, TNF-α, and BDNF levels were carried out using the Kruskal–Wallis test followed by Dunn’s multiple comparisons test for post hoc comparisons. Additionally, behavioral trajectories, classifications in the observing cage, and movement steps within the cage were examined through one-way analysis of variance (ANOVA) with post hoc evaluations performed via Tukey’s multiple comparisons test. For HIT, a two-way ANOVA was applied, accompanied by further post hoc pairwise comparisons adjusted using Tukey’s multiple comparisons test. A post hoc power analysis was conducted to validate the effect size. The behavioral outcomes are reported as mean ± SEM, with a *p*-value of less than 0.05 indicating statistical significance.

## 3. Results

The findings were systematically arranged as follows: Firstly, the implementation of the CUMS protocol led to a significant rise in anxiety levels and induced depression-like behaviors in monkeys. Secondly, the administration of TAK-653 was effective in alleviating these depression-like symptoms. Lastly, alterations in cortisol and inflammatory cytokine levels suggest potential underlying pathological mechanisms.

### 3.1. Effective Modeling of MDD in Monkeys

**Figure 2:**
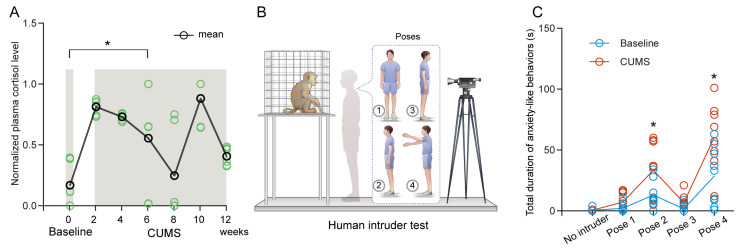
The increased anxiety levels following CUMS. (**A**) The normalized cortisol levels in plasma were measured before and after the CUMS intervention. The green circles represent the raw data, while the gray shaded regions denote the Baseline and CUMS conditions used for comparison. (**B**) A schematic representation of the HIT is provided. ① Profile pose: The intruder entered the room, standing 1 m away from the cage and maintained this distance for a duration of 2 min. ② Stare pose: The intruder then turned 90 degrees to face the monkey directly, maintaining eye contact for another 2 min without exiting the room. ③ Back pose: Next, the intruder turned 180 degrees to position themselves directly opposite the monkey for a continued period of 2 min while remaining present in the room. ④ Waving arm pose: Finally, the intruder rotated back towards the monkey and waved their arms for 2 min. (**C**) The cumulative duration of anxiety-like behaviors observed across the five poses is presented for baseline (blue) and after CUMS (red). (* *p* < 0.05, as determined by Tukey’s multiple comparisons test).

Prior to assessing the antidepressant effects of TAK-653 in NHP, it was imperative to establish a viable model for MDD. Consequently, the CUMS protocol was implemented across all three subjects over a span of three months. Given that exposure to stress typically elevates anxiety levels and that cortisol is a prominent biomarker for anxiety, the cortisol concentrations in all subjects were meticulously monitored biweekly. As depicted in Figure 2A, which presents the plasma cortisol levels before and throughout the CUMS duration, there was a noticeable rise in cortisol levels immediately following the initiation of CUMS, which remained elevated for the duration of the protocol despite some variations (Figure 2A). Following the completion of the CUMS exposure, the traditional HIT was employed to further assess the monkeys’ anxiety responses (Figure 2B). As anticipated, an increase in anxiety-like behaviors was recorded in those subjected to CUMS (Figure 2C). The analysis by two-factor ANOVA indicated that the varying time intervals (*F*(1.697, 27.15) = 38.4, *p* < 0.0001), along with the conditions before and after chronic stress (*F*(1, 16) = 10.68, *p* = 0.0048), had substantial impacts on the anxiety levels observed in the subjects, highlighting a significant interaction (*F*(4, 64) = 4.909, *p* = 0.0016). Additionally, Tukey’s multiple comparisons test revealed that the monkeys exhibited increased anxiety-like behaviors when confronted with strangers who were facing (Figure 2C, pose 2, adjusted *p* = 0.028) and gesturing with their arms (Pose 4, adjusted *p* = 0.0152).

**Figure 3:**
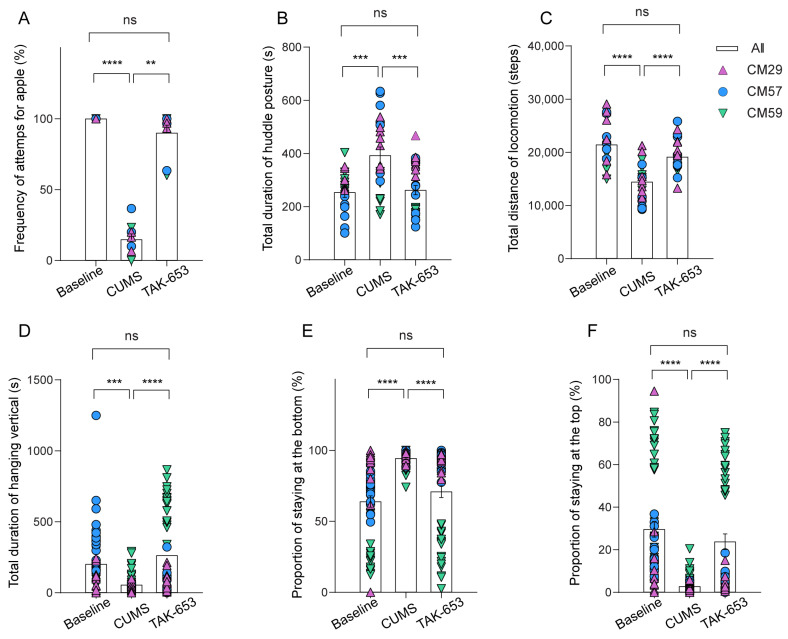
The antidepressant effects of TAK-653 on behaviors indicative of depression. (**A**) The frequency of attempts to access an apple over the three-day period. (**B**) The cumulative time spent in a huddled posture. (**C**) The overall duration of locomotor activity. (**D**) The total time spent hanging vertically. (**E**) The percentage of time spent at the bottom of the enclosure. (**F**) The percentage of time spent at the top of the enclosure. The three distinct colors and shapes correspond to three different subjects. (ns: *p* > 0.05, ** *p* < 0.01, *** *p* < 0.001, **** *p* < 0.0001, Tukey’s multiple comparisons test).

Furthermore, behavioral assessments revealed that following three months’ exposure to CUMS stimuli, the monkeys exhibited a diminished interest in apple treats (Figure 3A, *p* < 0.0001, Tukey’s multiple comparisons test), increased duration of huddling behavior (Figure 3B, *p* = 0.0005), reduced locomotion (Figure 3C, *p* < 0.0001), prolonged vertical hanging (Figure 3D, *p* = 0.0005), an elevated frequency of remaining at the bottom of the cage (Figure 3E, *p* < 0.0001), and a corresponding decrease at the top (Figure 3F, *p* < 0.0001). These behavioral manifestations are indicative of the fundamental symptoms associated with depression, thereby supporting the conclusion that the CUMS protocol is successful in generating models of MDD in this study.

### 3.2. TAK-653 Demonstrates Promising Antidepressant Effects

**Figure 4:**
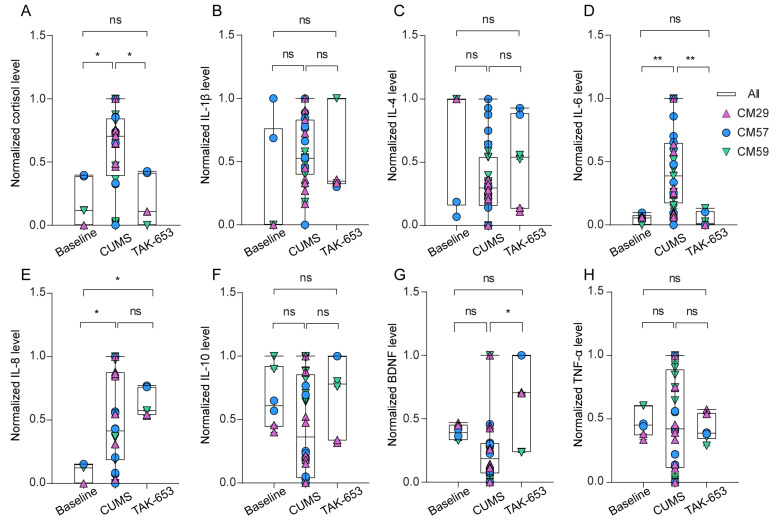
The variations in cortisol and inflammatory cytokines. (**A**) The normalized levels of cortisol. (**B**) The normalized levels of IL-1β. (**C**) The normalized levels of IL-4. (**D**) The normalized levels of IL-6. (**E**) The normalized levels of IL-8. (**F**) The normalized levels of IL-10. (**G**) The normalized levels of BDNF. (**H**) The normalized levels of TNF-α. The three distinct colors and shapes correspond to three different subjects. (ns: *p* > 0.05, * *p* < 0.05, ** *p* < 0.01, Tukey’s multiple comparisons test).

The objective of this study was to determine the potential of TAK-653 as an antidepressant candidate in NHP models. To achieve this, all subjects underwent a two-week treatment regimen with TAK-653, after which the same behavioral measurements employed previously were conducted. Results indicated that the monkeys exhibited a renewed interest in apple treats (Figure 3A, *p* = 0.0054), a reduction in huddling behavior (Figure 3B, *p* = 0.0003), and increased locomotion, as evidenced by their more active movements within the cage (Figure 3C, *p* < 0.0001). Additionally, there was a marked increase in the frequency of instances where monkeys hung at the top of the cage (Figure 3D, *p* < 0.0001), leading to less time spent at the bottom and a greater amount of time at the top (Figure 3E,F, *p* < 0.0001). Importantly, the performance observed following treatment was consistent with values recorded during the baseline period. Furthermore, the monkeys’ cortisol levels normalized post-treatment (Figure 4A, *p* = 0.0184). Collectively, these findings illustrate that TAK-653 treatment significantly alleviated depression symptoms in the monkeys.

### 3.3. Fluctuation of Inflammatory Cytokines Concentration Provides Hints for Mechanism of Action

The study subsequently examined the potential role of the immune system in the expression of depressive symptoms. A series of inflammatory cytokines were measured in plasma samples collected at baseline, at multiple time points during the CUMS phase, and after the treatment of TAK-653. To account for individual variability, the concentrations of these inflammatory cytokines were normalized per subject prior to calculating the overall averages, with the normalized levels illustrated in Figure 4. During the CUMS phase, there was a notable rise in cortisol (Figure 4A, *p* = 0.0184) accompanied by significant increases in IL-6 and IL-8 levels among the monkeys (Figure 4D, *p* = 0.0024; Figure 4E, *p* = 0.0254). In contrast, levels of IL-1β (Figure 4B), IL-4 (Figure 4C), IL-10 (Figure 4F), BDNF (Figure 4G), and TNF-α (Figure 4H) did not exhibit significant changes when compared to baseline measurements. Remarkably, following two weeks of TAK-653 treatment, only IL-6 levels reverted to baseline (Figure 4D, *p* = 0.0027), while IL-8 levels remained elevated as observed during the CUMS period (Figure 4E). This discrepancy may suggest that IL-6 and IL-8 have distinct kinetic responses and varying roles in the recovery process. Crucially, BDNF levels demonstrated an increase post-TAK-653 treatment (Figure 4G, *p* = 0.0395), supporting the hypothesis that TAK-653 stimulates AMPAR activity, thereby facilitating BDNF release. The other cytokines measured—IL-1β, IL-4, IL-10, and TNF-α—showed little variation throughout the different phases (Figure 4B,C,F,H), indicating their minimal involvement in the mediation of depression.

## 4. Discussion

This study initially showcases the effectiveness of CUMS in creating a model of depression-like behaviors in monkeys. It further evaluates the potential of TAK-653 as an antidepressant by analyzing its ability to alleviate depressive symptoms and explores the immune regulatory mechanisms by assessing inflammatory cytokine levels. Although the findings offer valuable insights for future studies, various challenges warrant additional discussion and exploration.

### 4.1. The CUMS Paradigm Is Effective in Modeling MDD in Monkeys

In the present research, the CUMS protocol was refined to establish a model that more accurately represents the intricate causes of MDD. Aligning with the results reported by Teng et al. [27], the current study demonstrates that exposure of cynomolgus monkeys to CUMS resulted in behaviors indicative of depression, such as anhedonia and decreased movement within their enclosures, as assessed by the AAT and motion tracking equipment. Additionally, following the implementation of the CUMS approach to model MDD, the monkeys displayed an increase in self-directed behaviors during the HIT, which is frequently utilized as a method to provoke anxiety-like responses in laboratory monkeys [31], signifying augmented anxiety-like tendencies. This is consistent with the finding that anxiety-related behaviors observed in rhesus monkeys were marked by an uptick in self-directed actions [43]. Thus, the monkeys subjected to the CUMS paradigm exhibited both depressive-like and anxiety-like behaviors. This finding is critical since anxiety and depression often manifest simultaneously in individuals with MDD, potentially sharing common underlying mechanisms [44]. Consequently, the biological and behavioral outcomes indicate that the CUMS paradigm employed in this study is effective in eliciting behavioral and physiological alterations in adult cynomolgus monkeys that parallel those of human MDD patients.

### 4.2. The Effect of TAK-653 as an Antidepressant

In this investigation, TAK-653 demonstrated a notable antidepressant effect in a non-human primate model of MDD. Post-treatment observations indicated an increased preference for apples among the monkeys, along with heightened movement within their cages, an enhanced standing posture, and prolonged time spent in the enclosure, all of which signify a marked improvement in depressive symptoms. Furthermore, throughout this study, none of the three monkeys exhibited any adverse effects, including excessive movement or seizures, following the treatment. This aligns with findings from previous research. In rat primary hippocampal neurons, TAK-653 exhibited minimal activity; however, it effectively enhanced AMPAR-mediated polysynaptic transmission in a more robust and efficient manner compared to other AMPA receptor modulators, particularly when AMPA induced AMPAR currents in a dose-dependent way [12]. Moreover, TAK-653 significantly boosted the phosphorylation levels of Akt, ERK, mTOR, and p70S6 kinase, as well as BDNF levels in primary cortical neurons under low AMPA concentrations, thereby activating the downstream signaling pathways typically engaged by ketamine [12].

In rat models of repeated-stress-based MDD, oral administration of TAK-653 at doses of 0.1 or 1 mg/kg was conducted once daily, with antidepressant-like outcomes assessed right after treatment and again one day later. After subchronic administration over seven and six days, TAK-653 exhibited potent antidepressant-like effects in these models, irrespective of circulating drugs or ketamine-induced psychomimetic behaviors [45]. Additionally, it improved sustained attention in underperforming rats during a five-choice serial reaction time task [41]. A three-chamber social sniff test with poly-I:C mice, which are used to model developmental disorders such as autism spectrum disorder and schizophrenia, revealed that TAK-653 could ameliorate social deficits. The influence of TAK-653 on working memory was assessed in monkeys utilizing the delayed match-to-sample paradigm and demonstrated considerable improvements in accuracy over a 16-s delay interval [41]. Furthermore, TAK-653 appeared to be safe and well-tolerated by healthy participants across both single and multiple rising dose studies without adverse effects. The CUMS paradigm effectively induced both behavioral and physiological changes in adult cynomolgus monkeys that are akin to those observed in MDD patients. Notably, TAK-653 also increased corticospinal excitability in healthy subjects as shown by transcranial magnetic stimulation, although neither of the two oral dosages impacted the resting motor threshold [40].

Collectively, these findings suggest that TAK-653 plays a role in modulating glutamatergic synaptic activity within the human brain. The safety profile of TAK-653 is substantial, boasting a 1017-fold margin after acute treatment regarding the area under the plasma concentration–time curve and a 419-fold margin concerning the peak plasma concentration [41]. In conclusion, TAK-653 demonstrates a minimal side effect profile and a wide safety margin, and it holds promise as an effective antidepressant. Additional research is imperative to elucidate the dose–response relationship in either NHP models or human subjects. Comprehensive toxicological evaluations must be conducted to guarantee safety. Furthermore, the establishment of stringent and well-defined endpoints is essential for the integrity of any clinical trial.

### 4.3. Potential Mechanism of Antidepressant Effects

Stress is a generalized physiological reaction to various demands placed on the body, with hyperactivation of the HPA axis emerging as a critical pathological indicator in individuals suffering from depression [46]. In the early phases of stress, the HPA axis is triggered, prompting the hypothalamus to secrete corticotropin-releasing hormone (CRH). This hormone stimulates the release of adrenocorticotropic hormone (ACTH) and cortisol, establishing a negative feedback loop aimed at dampening HPA axis activity. However, when stress becomes chronic, this feedback mechanism can fail, resulting in excessive cortisol production [47]. Accordingly, an elevated cortisol level in plasma was observed following the commission of CUMS in the current study.

The accumulating evidence implicating neuroinflammation in depression pathophysiology underscores a complex interplay between immune dysregulation, stress response, and neuroplasticity [48]. Proinflammatory cytokines such as IL-6 and TNF-α are elevated in MDD patients, reflecting a state of chronic low-grade inflammation that disrupts neuronal homeostasis [49,50]. These cytokines act as dual-edged mediators: while transient increases during acute stress may enhance synaptic plasticity via BDNF-mediated pathways, persistent elevation under chronic stress perpetuates neurotoxicity by promoting oxidative stress, microglial hyperactivation, and glutamate excitotoxicity [51,52]. The observed reduction in cortisol and IL-6 levels after TAK-653 treatment aligns with the hypothesis that dampening neuroinflammatory signaling may restore HPA axis homeostasis. Cortisol, a glucocorticoid with anti-inflammatory properties, paradoxically loses its regulatory efficacy under chronic stress due to glucocorticoid receptor (GR) resistance. TAK-653’s cortisol-lowering effect might mitigate GR desensitization, thereby enhancing endogenous feedback mechanisms to suppress IL-6 production. This is consistent with studies showing that IL-6 directly stimulates HPA axis activity [53], creating a vicious cycle of inflammation and cortisol dysregulation. The significant rise in BDNF levels post-treatment further supports TAK-653’s neuroprotective potential. BDNF is a critical modulator of synaptic plasticity [54], and its depletion under chronic inflammation correlates with hippocampal atrophy and prefrontal cortex dysfunction in MDD. However, the lack of change in IL-1β, IL-4, IL-10, and TNF-α implies cytokine-specific modulation rather than broad-spectrum anti-inflammatory action. For instance, IL-6 signals through both classical anti-inflammatory and trans proinflammatory pathways, and TAK-653 may preferentially target the latter via the membrane-bound IL-6 receptor. TAK-653’s apparent regulation of IL-6, cortisol, and BDNF aligns with emerging paradigms of depression as a disorder of neuroimmune crosstalk. By targeting cytokine-specific pathways, it may circumvent the limitations of conventional antidepressants, particularly in treatment-resistant populations with prominent inflammatory signatures. However, confirming these effects in larger, phenotypically stratified cohorts and integrating multi-omics approaches will be critical for validating its therapeutic potential [55,56].

### 4.4. Limitations

TAK-653 has demonstrated a significant capacity to mitigate anti-epileptic seizures across various doses, alongside exhibiting strong cognitive and antidepressant-like effects. This investigation lays a crucial foundation for the advancement of TAK-653 as an innovative antidepressant and further elucidates the role of the AMPA receptor in MDD. However, it is important to acknowledge certain limitations within this study.

Firstly, adhering to ethical standards [57], the experiment opted for the minimum sample size. The restricted number of monkeys prevented us from establishing varied dosing concentrations or conducting daily assessments of behavioral improvements post-dosing. Consequently, the optimal therapeutic concentration or the onset time for MDD improvement could not be determined. Secondly, to address safety concerns, a comprehensive dose/concentration response evaluation was not performed. Considering the potential, albeit modest, association between AMPA receptor PAMs (positive allosteric modulator) and an increased seizure risk, a therapeutic dose of 0.346 mg/kg was selected (equivalent to 1 mg/kg in rodents), as the anticipated mean maximum plasma concentration at this level is significantly lower than the concentration linked with partial seizures observed in rodent studies.

Moreover, it is well-documented that women are twice as likely to develop MDD compared to men. To mitigate the potential impact of sex hormones on our findings, this study exclusively utilized male cynomolgus monkeys. However, it remains uncertain whether these results can be extrapolated to females. Lastly, it is essential to recognize that the etiology of MDD is multifaceted, and a singular model may only represent one dimension of this complex disorder. Therefore, while the CUMS model has its merits, it may not encompass all the underlying causes of MDD.

## 5. Conclusions

The three-month CUMS protocol used in NHPs serves as a robust model for studying depressive-like behaviors. TAK-653 has shown promising antidepressant effects within the NHP model of MDD, highlighting its potential as a novel PAM of the AMPA receptor for MDD treatment.

## Figures and Tables

**Table 1:** The definition of each stressor in the CUMS paradigm.

Stressor	Definition
Fasting	Food was withheld for a continuous duration of 24 h, commencing at 7:00 a.m. and concluding at 7:00 a.m. the next day.
No water	Water access was deliberately limited for a continuous 12 h interval, precisely from 7:00 a.m. to 7:00 p.m., within a rigorously controlled experimental framework.
Noise	A sound-emitting apparatus was installed inside the experimental unit to produce continuous noise over a 12 h interval, precisely from 7:00 p.m. to 7:00 a.m. the next morning.
Stroboscope	A strobe light was installed one meter ahead of the monkey cages and activated to emit flashes at a frequency of 5 Hz continuously for a 12 h period.
Inescapable foot shocks	Electrodes were firmly attached to the plantar surfaces of the monkeys’ feet to administer electrical stimulation. Each stimulus involved a current intensity between 1.0 and 1.25 mA, delivered for a duration of 3 to 5 s, followed by a 10 s pause before the next stimulus. This sequence was systematically repeated three to four times.
Audiovisual stimulation	This presentation delivers a serious and detailed audiovisual depiction of the innate behavioral traits of large carnivores and raptors, encompassing actions such as roaring, running, and the pursuit and attack of prey.
Spatial restriction	The adjustable lever inside the cage was set to restrict the monkey’s range of movement, allowing just enough room for the monkey to turn around.

**Table 2:** Anxiety-related behaviors in the HIT paradigm.

Behavior	Definition
Freezing	Remaining completely still in the same posture for a duration exceeding two seconds.
Skin scratching	Repetitive motion of the fingers or toes moving across the fur.
Yawning	Open mouth and yawn.
Displaying signs of fear	The act of baring one’s canines, accompanied by widened eyes and an open mouth, serves as a deliberate display intended to intimidate others.
Retreating to the bottom of the cage	A swift withdrawal to the base of the cage, ensuring that no fewer than three limbs maintain contact with the cage floor.
Slow movements	Displaying at least three successive slow steps.
Shaking the cage	Firmly grasp the cage and shake it with force.
Swaying	Maintains an upright posture while swiftly oscillating from side to side.
Grooming	Grooming hair or skin.
Lip smacking	Swift and silent lip smacking.

**Table 3:** The definitions of distinct behaviors of monkeys housed in individual cages.

Behavior	Definition
Huddling posture	The position in which the head is situated between the shoulders and held lower than them while the individual is awake.
Locomotion	Moving and walking in the cage
Stereotypical behaviors	A consistent and recurring behavioral pattern that manifests no fewer than three times within a short timeframe.
Environmental responses	Responses to novel environment, including activities such as exploration and feeding.
Climbing	Climbing to the side walls or the top of the cage.
Self-grooming	Self-grooming
Ball play	Engaging in play with a ball that is suspended outside the confines of the cage.
Observing	Gazing beyond the confines of the cage to examine the surrounding environment.
Sitting	Remaining still, either seated or reclining at the base of the cage.

## Data Availability

The datasets presented in this article are not readily available because the data are part of an ongoing study. Requests to access the datasets should be directed to the corresponding author.

## Abbreviations

MDD	Major Depressive Disorder
AMPAR	α-amino-3-hydroxy-5-methyl-4-isoxazolepropionic acid receptors
NMDAR	N-methyl-D-aspartate receptor
BDNF	Brain-derived neurotrophic factor
NHP	Non-human primate
HPA	Hypothalamic-pituitary-adrenal
CUMS	Chronic unpredictable mild stress
AAT	The Attempt for Apple Test
HIT	The Human Intruder Test
